# Food Addiction Symptoms and Amygdala Response in Fasted and Fed States

**DOI:** 10.3390/nu11061285

**Published:** 2019-06-06

**Authors:** Kirrilly M. Pursey, Oren Contreras-Rodriguez, Clare E. Collins, Peter Stanwell, Tracy L. Burrows

**Affiliations:** 1 Faculty of Health and Medicine, The University of Newcastle, University Drive, Callaghan, NSW 2308, Australia; kirrilly.pursey@newcastle.edu.au (K.M.P.); Clare.Collins@newcastle.edu.au (C.E.O.); peter.stanwell@newcastle.edu.au (P.S.); 2 Priority Research Centre for Physical Activity and Nutrition, The University of Newcastle, University Drive, Callaghan, NSW 2308, Australia; 3 Department of Psychiatry, Bellvitge Biomedical Research Institute (IDIBELL), and CIBERSAM, 08907 Barcelona, Spain; orencoro@gmail.com

**Keywords:** Food addiction, Yale Food Addiction Scale, functional magnetic resonance imaging, basolateral amygdala

## Abstract

Few studies have investigated the underlying neural substrates of food addiction (FA) in humans using a recognised assessment tool. In addition, no studies have investigated subregions of the amygdala (basolateral (BLA) and central amygdala), which have been linked to reward-seeking behaviours, susceptibility to weight gain, and promoting appetitive behaviours, in the context of FA. This pilot study aimed to explore the association between FA symptoms and activation in the BLA and central amygdala via functional magnetic resonance imaging (fMRI), in response to visual food cues in fasted and fed states. Females (*n* = 12) aged 18–35 years completed two fMRI scans (fasted and fed) while viewing high-calorie food images and low-calorie food images. Food addiction symptoms were assessed using the Yale Food Addiction Scale. Associations between FA symptoms and activation of the BLA and central amygdala were tested using bilateral masks and small-volume correction procedures in multiple regression models, controlling for BMI. Participants were 24.1 ± 2.6 years, with mean BMI of 27.4 ± 5.0 kg/m^2^ and FA symptom score of 4.1 ± 2.2. A significant positive association was identified between FA symptoms and higher activation of the left BLA to high-calorie versus low-calorie foods in the fasted session, but not the fed session. There were no significant associations with the central amygdala in either session. This exploratory study provides pilot data to inform future studies investigating the neural mechanisms underlying FA.

## 1. Introduction

There is increasing scientific interest in the possible role of “food addiction” (FA) underlying particular patterns of overeating, dietary relapse and weight gain in vulnerable individuals. Neuroimaging techniques, such as functional magnetic resonance imaging (fMRI), have provided insight into this phenomenon in humans. Visual food cues and consumption of palatable, energy-dense foods have been shown to activate reward-related brain circuits in humans in a similar way to substances of abuse [1]. Despite accumulating evidence supporting FA as a phenomenon in preclinical [2] and behavioural research [3], few studies have investigated the potential underlying neural substrates in humans. Many studies have used obesity as a proxy for addictive-like eating in lieu of a recognised assessment tool for FA, such as the Yale Food Addiction Scale (YFAS) [4]. Using obesity as a proxy for FA could result in inconsistent neuroimaging findings as it is unclear as to the proportion of participants truly affected by addictive-like eating in these samples.

In one neuroimaging study using a recognised FA assessment tool, YFAS symptoms were associated with greater activation in response to a visual milkshake cue in brain areas encoding the reward value of foods and craving (amygdala, anterior cingulate cortex (ACC), medial orbitofrontal cortex (OFC), and the dorsolateral prefrontal cortex (DLPFC)) in young females [5]. In another study, while main effects were found activation in the amygdala, OFC, nucleus accumbens and inferior frontal cortex in response to the taste of sugar-sweetened beverages, no relationships were observed between YFAS symptoms and neural response in male and female adolescents [6]. The divergence in previous studies may be related to the limited range of food cues used, which have often been restricted to sweetened beverages [5,6]. These previous studies have also not studied participants in different motivational states (i.e., fasted and fed), which is important given the differences in responsivity in reward-related networks in these states [7,8].

The amygdala has been implicated in previous FA neuroimaging research [5] and plays a role in regulating the hedonic impact of salient stimuli and coordinating appetitive behaviours, with studies reporting selective sensitivity of the amygdala to food cues in the fasted state [9,10]. The amygdala has also been shown to integrate interoceptive states and sensory cues along the ventral visual stream [9] and has been implicated in drug cue reactivity and drug craving [11,12]. While the amygdala may play an important role in the context of FA, no studies have explored the potential role of distinct subregions of the amygdala. The basolateral amygdala (BLA) is of particular interest as it has been shown to drive external cues to the hypothalamic feeding centres in both humans and rats [13,14], consistent with the role in processing high-level sensory input and stimulus-value associations in humans [15]. In animal studies, the BLA has been implicated in reward-seeking behaviours in response to food-related stimuli [16] and relapse to food seeking [17]. In the satiated state, the BLA has also been associated with eating in the absence of homeostatic needs in rats [18] as well as predicting weight gain susceptibility in males and females [13]. In addition, the central amygdala has been reported to have a role in increasing reward saliency, modulating food consumption and promoting appetitive behaviours in mice [19]. While these previous studies of the BLA and central amygdala were not conducted in FA populations specifically, these findings suggest that there is a need to study the subregions of the amygdala in relation to FA in different motivational states.

This pilot study aimed to explore the association between YFAS assessed FA symptoms and activation in the central and BLA, assessed via functional MRI, in response to visual food cues in fasted and fed states. It was hypothesised that FA symptoms would be associated with greater activation in the BLA in response to high-calorie vs low-calorie foods in both the fasted and fed states, based on the findings of previous neuroimaging research [7,9,10].

## 2. Materials and Methods

### 2.1. Participants

Australian females aged 18–35 years were recruited to the current study from an existing pool of participants who had completed an online FA survey, which aimed to determine FA prevalence and associations with dietary intake in Australian adults [20]. At the end of the survey, participants could exit the survey with no further contact or could volunteer to be recontacted for future research. Full details regarding the FA survey are published elsewhere [20]. This study was approved by the University of Newcastle Human Research Ethics Committee (Approval number H-2012-0419) and was conducted in accordance with the ethical standards of the 1964 Helsinki Declaration.

Participants were eligible for the current study if they were female, aged 18–35 years, lived within a one-hour proximity to the imaging facility (Newcastle, NSW, Australia) and elected to be contacted for future research at the end of the online survey. Eligibility for this pilot study was restricted to females only in order to reduce potential inter-person variation in appetite and neural activation to visual food cues associated with sex-related differences [21]. Seventy-seven participants from the original survey volunteered for future research and were recontacted via email to participate in the fMRI component of the study. Of those recontacted, 35 responded that they were interested in participating in the fMRI study and were screened via telephone. Exclusion criteria for the current study included pregnancy, body mass >150 kg due to weight limitations for the MRI scanner, contraindications to MRI, left handedness, pre-existing medical or Axis 1 disorders, disordered eating behaviour, medications affecting appetite, history of substance abuse or head injury with loss of consciousness, allergy to any beverage ingredients, risk of adverse medical events as a result of fasting (e.g., diabetes), or inability to refrain from cigarette smoking. Seventeen participants did not meet the inclusion criteria for the current study, and five participants were not able to be contacted for screening, resulting in a final sample of thirteen participants. Written informed consent was obtained from all participants in the current study.

### 2.2. Procedures

**Figure 1:**
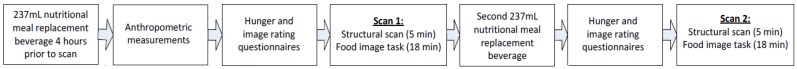
Flow diagram of the study procedure.

The study procedure is outlined in Figure 1. Participants attended a single session where they underwent two fMRI scans of the brain, the first in the fasted state and the second in the fed state. Four hours prior to the first scan, participants consumed a standardised pre-fast meal (237 mL Ensure Plus; 1485 kJ/355 kcal). Participants then fasted for four hours, excluding water, to capture the hunger-state experienced when approaching the next meal. Participants were instructed to avoid caffeinated and alcoholic beverages, and smoking for twelve hours prior to the scan. Compliance with the pre-scan protocol was checked via verbal self-report upon arrival at the imaging facility prior to scanning. The session included anthropometric measurements, a demographic survey, hunger and image ratings, and the first of two fMRI scans. Following the first scan, participants drank a second Ensure Plus drink with compliance monitored by the research team. Participants again completed the hunger and image ratings before undertaking a second fMRI scan approximately 45 min following the completion of the second meal replacement beverage. Participants were not able to be scanned at the same time of day due to scheduling at the imaging facility.

### 2.3. Measures

Demographics: Demographic data including sex, age, indigenous status, marital status and highest qualification were collected. Current and previous dieting history [22] was also assessed.

Yale Food Addiction Scale: The YFAS is a 25-item questionnaire which assesses addictive-like eating according to the diagnostic criteria for substance dependence. The YFAS has been shown to have adequate psychometric properties [4]. The tool includes two scoring options, a symptom score from 0–7 based on the DSM criteria for substance dependence, as well as a diagnosis of FA if ≥3 symptoms are reported as well as clinical impairment or distress. Cronbach α for the current sample was 0.93.

Anthropometrics: Height was taken using a BSM370 stadiometer, while weight and body composition (% body fat, fat free mass) was assessed using the InBody720 bioelectrical impedance analyser (Biospace, Seoul, Korea) using a standardised protocol. Body mass index (BMI) was subsequently calculated.

Hunger and Image Ratings: Participants completed a set of ratings related to hunger and appetite, as well as rating a subset of images shown to them in the MRI scanner (*n* = 20; *n* = 10 high-calorie, *n* = 10 low-calorie), using a 10 cm visual analogue scale. Participants completed the set of ratings in the fasted state at baseline (prior to Scan 1) and in the fed state (prior to Scan 2). Hunger and appetite ratings included self-reported (i) hunger, (ii) satiety, (iii) fullness and (iv) prospective food consumption [23]. For the image ratings, participants were asked to rate the (i) appeal of the food, (ii) desire to eat the food, (iii) whether the food increased their appetite and (iv) perceptions of prospective food consumption amount related to each of the food images.

### 2.4. Imaging Paradigm

Food images were chosen from a standardised database [24] and foods representative of the Australian food environment. Images were matched for brightness, complexity, resolution and size. Two groups of images were created according to nutrient composition, (i) appetising high-calorie foods (e.g., chocolate, chips, pizza, ice cream) and (ii) low-calorie foods (fruits and vegetables). The selected images were informed by the foods commonly reported in the original FA online survey. Food images were piloted on a sample of six university students independent of the study sample (mean age 27 years) regarding recognisability, familiarity and appeal. Those foods with low pilot ratings for recognisability and familiarity were excluded (*n* = 27). A final sample of 150 single food images were selected for the study including *n* = 75 high-calorie and *n* = 75 low-calorie foods. Nutritional composition of the food images can be found in Table S1.

Visual stimuli were presented in a block design format using Presentation software (Neurobehavioral Systems, Inc, Berkley, USA) and NordicNeuroLab sync box (NordicNeuroLab AS, Bergen, Norway), and consisted of two 18 min 20 s runs. Each run consisted of 15 epochs each of (i) low-calorie foods, (ii) high-calorie foods and (iii) fixation cross. Within each 20 s epoch of food images, five images were presented for four seconds each. A 20 s central fixation cross was presented as a control, and each epoch was separated by a four second blank screen. Each run consisted of 440 volumes over the scanning period. Images were presented onto an MRI compatible screen via a projector, and viewed via a mirror attached to the head coil. Standardised instructions to remain still focus on the visual stimuli were provided by the radiographer during the scan.

### 2.5. Imaging Data Collection

Structural and task-related functional scans were acquired using a Siemens 3-tesla MRI (Siemens AG, Germany). Structural images were acquired with a three-dimension (3D) magnetisation-prepared rapid gradient-echo (MP-RAGE) sequence with the following parameters: TE = 3.5 ms, TR = 2 s, 7° excitation flip angle, 160 slices with 1mm isotropic resolution. Task-related functional MR images were acquired using a T2*-weighted gradient-echo echo planar imaging (EPI) pulse sequence. The parameters were TE = 24 ms, TR = 2.5 s, 90° excitation flip angle, 47 axial slices 3 mm thick with a 0 mm interslice gap, in-plane resolution of 3 × 3 mm, covering from vertex to cerebellum. Axial slices were angled to the anterior cranial floor for to limit distortions that may impact on imaging of the orbito-frontal region.

### 2.6. Statistical Analysis

Participant characteristics, hunger and image ratings were analysed descriptively using Stata13. Differences in hunger and image ratings between the first and second scan were assessed using paired t-tests. Task-related functional MR images were processed and analysed using MATLAB version R2017a (The MathWorks Inc, Natick, Mass, USA) and Statistical Parametric Software (SPM12; The Welcome Department of Imaging Neuroscience, London, UK). Preprocessing steps involved motion correction, spatial normalization and smoothing using a Gaussian filter (FWHM 8 mm). The realigned functional sequences were coregistered to each participant’s anatomical scan, which had been previously coregistered and normalized to the SPM-T1 template. Normalization parameters were then applied to the coregistered functional images, which were then resliced to a 2 mm isotropic resolution in Montreal Neurological Institute (MNI) space.

First-level (single-subject) SPM contrasts images were estimated for each of the fasted and fed scans for the following task main effects of interest: high-calorie > low-calorie foods, and low-calorie > high-calorie foods. Changes in brain activation between sessions were computed by the creation of the following contrasts: (Fasted scan (high-calorie > low-calorie foods) – Fed scan (high-calorie > low-calorie foods)) and (Fed scan (high-calorie > low-calorie foods) – Fasted scan (high-calorie > low-calorie foods)). The same contrasts were created to explore the between session effects on the activation of low-calorie > high calorie foods. Three regressors were used in these analyses to model conditions separately corresponding to high-calorie and low-calorie foods, and the fixation cross. Furthermore, to correct for subtle in-scanner movements from volume-to-volume scans, we identified the outlier scans present in the realigned task-related functional scans as determined using the CONN toolbox (Whitfield-Gabrieli and Nieto-Castanon, 2012). For each participant, the actual removal of outlier scans was completed by entering the subject-specific variables identifying the outlier scans (i.e., one regressor per outlier) in the first-level models as covariates of no interest, so these outlier scans were removed from these and subsequent analyses. We excluded one subject that had 19.8% of outlier scans. A hemodynamic delay of 4 s and a high-pass filter (1/120 Hz) were considered. The resulting first-level contrasts images were then carried forward to subsequent second-level random-effects (group) analyses. One-sample t-tests were used to assess the main task effects in the fasted and fed sessions. Multiple regression models were used to assess associations between FA symptoms and whole-brain activation under the main task effects in the fasted and fed sessions, and the change between sessions. These analyses were controlled for the subject-specific number of outlier scans, BMI, hunger and dieting when the association with FA symptoms was explored. Results were considered significant when surviving a *p* > 0.001 and a determined cluster-extent using Monte Carlo simulations implemented in the AlphaSim thresholding approach [25] (94 voxels and 135 voxels for the fasted and fed sessions, respectively incorporating a 2×2×2mm grey matter mask of 128,190 voxels). However, we specifically tested for associations between FA symptoms and the activation of the basolateral and central amygdala using bilateral masks and small-volume correction procedures in the multiple regression models. To that end, we defined bilateral basolateral and centromedial amygdala masks comprising 3.5 mm radial spheres cantered at left basolateral (x = −26, y = −5, z = −23) and right BLA (x = 29, y = −3, z = −23), and the left centromedial amygdala (x = −19, y = −5, z = −15) and right centromedial amygdala (x = 23, y = −5, z = −13) following previous research [26,27].

## 3. Results

Participant characteristics can be found in Table 1. The mean YFAS symptom score for the group was 4.1 ± 2.2 (range 1–7), with six participants classified as having a YFAS FA diagnosis. Hunger and image ratings are presented in Table 2. Hunger ratings were not significantly different between the fasted and fed scans (*p* = 0.13); however, ratings of fullness and satisfaction increased, and prospective food consumption decreased (all *p* < 0.05). Image ratings were significantly reduced from the fasted to fed scans (*p* < 0.05). FA symptoms were not associated with hunger or image ratings (*p* > 0.05)

### Brain Activation and Association with FA Symptoms

**Figure 2:**
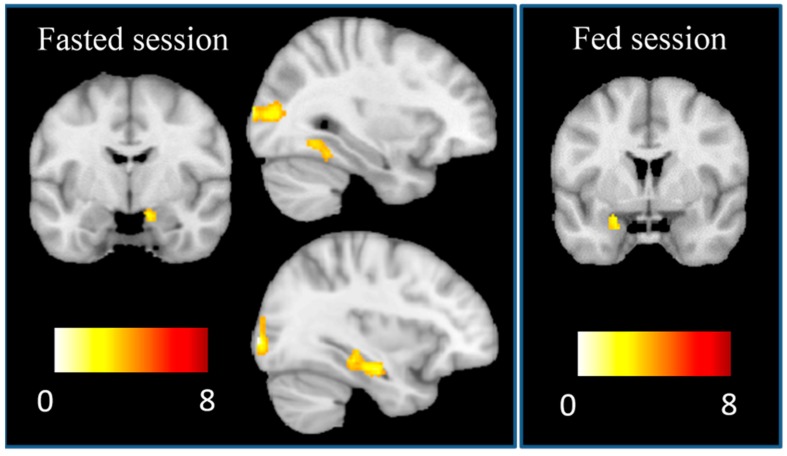
Increased brain activation to “high-calorie vs low-calorie” foods during the fasted and the fed sessions. Right side of the figure corresponds to the right hemisphere in the coronal views. In sagittal views, the upper figure corresponds to the right hemisphere, whereas the lower figure corresponds to the left hemisphere. Colour bar displays t-values for the comparison of activation in response to high-calorie vs low-calorie food cues.

Visual cues of high-calorie vs low-calorie foods activated a cluster in the right amygdala, the left hippocampus, the fusiform gyrus, and in bilateral occipital cortex in the fasted session, and the left amygdala in the fed session (Table 3, Figure 2). No significant activations were recorded in the contrast low-calorie vs high-calorie foods in the fasted and fed sessions.

**Figure 3:**
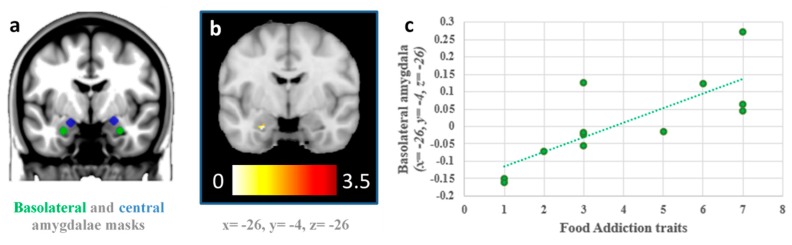
Association between the activation of the left basolateral amygdala and food addiction traits, (**a**) location of the basolateral (green) and central (blue) amygdala seeds, used as a mask for small-volume corrections; (**b**) left basolateral amygdala significantly associated with food addiction traits during the fasted session (*p*_SVC-FWE <0.05_ = 0.042); (**c**) scatter plot represents the correlation between the change in the activation of the left basolateral amygdala from the fasted to the fed sessions (y-axis) and food addiction traits (x-axis) (x = −26, y = −4, z = −26, t = 3.77, *p*_SVC-FWE<0.05_ = 0.027).

No significant associations with FA symptoms were found using whole-brain corrections, but a significant association between the FA symptoms and higher activation of the left BLA to high-calorie vs low-calorie foods in the fasted session (x = −26, y = −4, z = −26, t = 3.45, 11 voxels, *p*_SVC-FWE<0.05_ = 0.042) were identified using small-volume correction procedures. Importantly, this association was found regardless of the body mass index of the participants, and it also remained statistically significant after controlling for hunger reported by the participants during the fasted session and current dieting status. No significant associations were identified between FA symptoms and the activation of the central amygdala in the fasted session, and the activation of the basolateral and central amygdala in the fed session (all *p*_SVC-FWE_ >0.05). The change in the magnitude of activation from the fasted to the fed sessions of the left BLA showed a significant association with FA symptoms, controlling for BMI (x = −26, y = −4, z = −26, t = 3.77, 8 voxels, *p*_SVC-FWE<0.05_ = 0.027) (Figure 3).

## 4. Discussion

This is the first study to use fMRI to investigate activation in distinct subregions of the amygdala in response to visual food cues in relation to FA symptoms in both fasting and fed states. In line with the hypothesis, activation in the BLA in response to high-calorie foods versus low-calorie foods was found to be associated with higher FA symptoms in the fasted state, which is consistent with previous research [7,9,10]. Furthermore, participants with greater FA symptoms showed higher activation in the BLA in the fasted than in the fed scans to the sight of high-calorie vs low-calorie foods. One possible mechanism consistent with this is that the amygdala boosts activation in the ventral stream [9,28], increasing food salience in the hunger state. Individual differences in BLA response in the hunger state to appetizing, high-calorie foods may therefore be associated with susceptibility to overeating.

The findings of the current study suggest that those with higher FA symptoms may be vulnerable to environmental food cues of energy-dense, nutrient-poor, high-calorie food when hungry compared to those with lower FA symptoms. This may lead those vulnerable individuals to greater desire to eat, hence leading to food seeking and consumption, which is consistent with previous research [16,17]. It is possible that FA may play a mediating role in non-homeostatic eating, dietary relapse and other longer-term issues such as weight gain. This may be an important consideration in future interventions targeting FA, suggesting a regular meal pattern that uses strategies to avoid long periods of fasting may be warranted. This may also suggest that further consideration to reducing environmental food cues (e.g., food advertisements) in treatment approaches may be warranted for those vulnerable to addictive-like eating.

The second hypothesis was not supported, with no significant associations found between FA symptoms and BLA activation in the fed state. This is divergent previous work investigating the role of the amygdala in eating in the absence of hunger and weight gain susceptibility [13]. However, the study by Sun and colleagues [13] did not recruit a sample to investigated FA, in contrast to the current study. These differences may also be due to the study samples (i.e., inclusion of males in the previous study) and use of different food cues (i.e., taste vs sight). Alternatively, this may be related to the standardised meal not significantly reducing self-reported hunger from baseline in the current study, although fullness was increased, and prospective food consumption was decreased. Of note, the standardised meal would have contributed a relatively small proportion of total daily energy intake (TEE) for participants (<30%). Future studies should therefore consider the use of a standardised meal with greater contribution to %TEE.

The strengths of the current study include the use of a recognised assessment tool for FA and investigation of participants in fasted and fed states. Food images were informed by previous research and selected from a standardised database [24], which was found to be a limitation of previous studies [21]. This current exploratory study is limited by the small sample size and inclusion of females only. Additionally, participants could not be scanned at the same time of day, however, a standardised meal replacement was provided to reduce inter-individual variation. Self-reported hunger was not significantly different between the fasted and fed states, however, other measures of appetite were significantly reduced. Future studies should consider standardised meals with greater calorie content, to ensure hunger is significantly different between the two conditions. As the scans were not conducted in a counterbalanced order, it is possible that the lack of associations may be attributed to a habituation effect. A further limitation is the lack of control condition in the cue reactivity task, and the addition of a control condition should be considered in future studies. Although participants were asked about disordered eating behaviours during the screening process, a clinical interview to identify eating disorder was not included in the baseline assessment. Future studies should use a clinical eating disorder assessment to better control for underlying disordered eating. This study used the original YFAS tool as the updated YFAS 2.0 tool had not been released at the time of the study. Future imaging studies should consider the use of the YFAS 2.0, which aligns with the DSM-5 criteria [29]. The current study also investigated associations between neural activation and FA symptoms, however, future studies may consider analysing according to the dichotomous YFAS diagnosis and in those with higher FA symptoms to better understand addictive-like eating.

## 5. Conclusions

This study demonstrates that activation of the BLA, which has been linked to reward-seeking behaviours and susceptibility to weight gain, was associated with FA symptoms in the fasted state. This study provides pilot data to inform future studies with larger sample sizes investigating the neural mechanisms associated with FA.

## Figures and Tables

**Table 1:** Participant characteristics at baseline (*n* = 12).

Characteristic	Total Sample
**Age** (years)	24.1 ± 2.6 (range 21–29)
**Aboriginal or Torres Strait Islander background *n*** (%)	0 (0)
**Highest level of education *n*** (%)	
Higher school certificate	2 (17)
Diploma	2 (17%)
University degree	8 (67%)
**Body mass index (BMI)** (kg/m^2^)	27.4 ± 5.0 (range 21.7–39.0)
**Body fat** (%)	32.5 ± 9.8 (range 18.7–50.1)
**Symptom score**	4.1 ± 2.2 (range 1–7)
**Current dieting *n*** (%)	6 (50)
**Previous dieting *n*** (%)	12 (100)

Data is presented as mean ± SD unless otherwise specified.

**Table 2:** Hunger, appetite and image ratings in the fasted and fed states.

	Rating		*p*
	Fasted Scan	Fed Scan	
**Hunger and appetite ratings**			
Self-reported hunger	5.8 ± 1.9	4.2 ± 3.0	0.13
Self-reported satisfaction	3.1 ± 2.4	5.4 ± 2.6	0.03
Self-perceived fullness	2.1 ± 2.3	5.8 ± 3.1	0.003
Prospective food consumption amount	7.2 ± 1.8	4.8 ± 2.7	0.02
**Image ratings**			
Appeal of food	6.0 ± 1.6	4.5 ± 1.3	0.02
Desire to eat food	5.2 ± 1.7	3.8 ± 1.2	0.03
Effect of food in increasing appetite	4.5 ± 1.6	3.1 ± 1.1	0.02
Prospective consumption amount of food depicted in image	5.2 ± 1.3	3.2 ± 1.0	<0.001

Ratings completed using a 10 cm visual analogue scale. Results are presented as mean ± SD.

**Table 3:** Brain regions showing significant activation in response to the sight of high-calorie vs low-calorie foods in the fasted and fed sessions.

Brain Activation	Coordinates (x, y, z)	t-Value	CS
**Fasted session**			
Amygdala	14, −6, −18	6.1	111
Hippocampus	−36, −18, −16	6.7	243
Fusiform gyrus	32, −46, −16	5.2	127
Occipital cortex	42, −88, 6	8.3	434
	−34, −94, 2	7.9	165
**Fed session**			
Amygdala	−24, 14, −20	7.5	314

Coordinates are given in Montreal Neurological (MNI) Atlas space. The results for the fasted session surpassed a *p* < 0.001 and a cluster size (CS) of 94 voxels and 135 voxels, for the fasted and fed session, respectively. The t-values refer to the comparison of the activation of each of the listed brain regions to the high-calorie vs low-calorie food images.

## Supplementary Materials

The following are available online at https://www.mdpi.com/2072-6643/11/6/1285/s1, Table S1: Nutritional composition of food image groups per 100 g.
