# Supplementary material for: Food Addiction Symptoms and Amygdala Response in Fasted and Fed States
Source: Nutrients. 2019 Jun 6;11(6):1285. doi: 10.3390/nu11061285 (PMC6628069; doi:10.3390/nu11061285)
Supplement: Supplementary file 1 [file nutrients-11-01285-s001.pdf]

Table S1. Nutritional composition of food image groups per 100 g.

	**High-Calorie Food Group**	**Low-Calorie Food Group**
Energy (kJ) *	1440.6 ± 552.1	156.1 ± 79.5
(kCal) *	344.3 ± 132.0	37.3 ± 19.0
Protein (g) *	7.3 ± 3.7	1.3 ± 1.0
Fat (g) *	16.6 ± 9.9	0.2 ± 0.1
Carbohydrate (g) *	40.3 ± 20.0	6.2 ± 5.1

*= $p < 0.001$ high-calorie vs low-calorie. Data is presented as mean ± SD.
